# A longitudinal analysis of the relationship between emotional symptoms and cognitive function in patients with major depressive disorder

**DOI:** 10.1017/S0033291725001011

**Published:** 2025-05-02

**Authors:** Jingjing Zhou, Jinjie Xu, Zizhao Feng, Rui Liu, Le Xiao, Ruinan Li, Xiaoya Li, Xueshan Zhang, Jing Liu, Yuan Feng, Jia Zhou, Gang Wang

**Affiliations:** 1 The National Clinical Research Center for Mental Disorders & Beijing Key Laboratory of Mental Disorders, Beijing Anding Hospital, Capital Medical University, Beijing, China; 2 Advanced Innovation Center for Human Brain Protection, Capital Medical University, Beijing, China

**Keywords:** depressive symptoms, major depressive disorder, objective cognition, social functioning, subjective cognition, cohort study, cognitive impairment, Hamilton Depression Rating Scale (HAMD-17), Quick Inventory of Depressive Symptomatology-Self Report (QIDS-SR16), Chinese Brief Cognitive Test (C-BCT), Perceived Deficits Questionnaire for Depression-5 (PDQ-D5), Sheehan Disability Scale (SDS)

## Abstract

**Background:**

The relationship between emotional symptoms and cognitive impairments in major depressive disorder (MDD) is key to understanding cognitive dysfunction and optimizing recovery strategies. This study investigates the relationship between subjective and objective cognitive functions and emotional symptoms in MDD and evaluates their contributions to social functioning recovery.

**Methods:**

The Prospective Cohort Study of Depression in China (PROUD) involved 1,376 MDD patients, who underwent 8 weeks of antidepressant monotherapy with assessments at baseline, week 8, and week 52. Measures included the Hamilton Depression Rating Scale (HAMD-17), Quick Inventory of Depressive Symptomatology-Self Report (QIDS-SR16), Chinese Brief Cognitive Test (C-BCT), Perceived Deficits Questionnaire for Depression-5 (PDQ-D5), and Sheehan Disability Scale (SDS). Cross-lagged panel modeling (CLPM) was used to analyze temporal relationships.

**Results:**

Depressive symptoms and cognitive measures demonstrated significant improvement over 8 weeks (*p* < 0.001). Baseline subjective cognitive dysfunction predicted depressive symptoms at week 8 (HAMD-17: β = 0.190, 95% CI: 0.108–0.271; QIDS-SR16: β = 0.217, 95% CI: 0.126–0.308). Meanwhile, baseline depressive symptoms (QIDS-SR16) also predicted subsequent subjective cognitive dysfunction (β = 0.090, 95% CI: 0.003-0.177). Recovery of social functioning was driven by improvements in depressive symptoms (β = 0.384, *p* < 0.0001) and subjective cognition (β = 0.551, *p* < 0.0001), with subjective cognition contributing more substantially (R^2^ = 0.196 vs. 0.075).

**Conclusions:**

Subjective cognitive dysfunction is more strongly associated with depressive symptoms and plays a significant role in social functioning recovery, highlighting the need for targeted interventions addressing subjective cognitive deficits in MDD.

## Introduction

Major depressive disorder (MDD) is a prevalent condition that affects approximately 280 million individuals globally (World Health Organization, 2023). It is a leading cause of disability worldwide and significantly contributes to the global burden of disease (GBD 2019 Diseases and Injuries Collaborators, 2020). A study on first-episode drug-naive patients with MDD found that 13.7% of the patients exhibited suicide attempts within 1 month of the study (Li et al., 2024). Annually, more than 700,000 individuals die by suicide due to depression (World Health Organization, 2023).

Cognitive impairment is a significant manifestation of MDD; however, its relationship with depressive symptoms remains unclear. Current diagnostic criteria, as outlined in the Diagnostic and Statistical Manual of Mental Disorders, Fifth Edition (DSM-5), recognize cognitive disturbances as associated features of MDD. A meta-analysis (Rock, Roiser, Riedel, & Blackwell, 2014) revealed significant moderate cognitive deficits in executive function, memory, and attention in patients with MDD; it also found that these cognitive impairments are fundamental aspects of the disorder, rather than mere epiphenomena secondary to low mood symptoms. Although cognitive symptoms are traditionally thought to be influenced by the depressive state, research indicates that these deficits can persist even in remitted states (Preiss et al., 2007; Rock et al., 2014), negatively impacting patients’ overall functionality (Evans, Iverson, Yatham, & Lam, 2014). Our previous study found that difficulty with concentration and decision-making was the core residual symptom of MDD and was associated with poorer social functioning, increased family burden, and lower life satisfaction (Zhou et al., 2024). However, to date, the question of whether they represent independent or concomitant symptoms remains unresolved.

Cognitive function can be evaluated through subjective self-assessments or objective tests. However, studies have identified significant discrepancies and weak correlations between subjective and objective cognitive measures in patients with MDD (Miller, 1975; Serra-Blasco et al., 2019). This discrepancy may be partially attributed to findings that emotional symptoms adversely affect subjective cognition but not objective cognition (Srisurapanont, Suttajit, Eurviriyanukul, & Varnado, 2017). This supports the hypothesis that depression severity contributes to negative cognitive bias, and the relationship between emotional symptoms and cognitive function may differ depending on whether it is subjectively or objectively assessed. Restoring social functioning is a key indicator of recovery from depression (Oluboka et al., 2018). While it has been traditionally assumed that alleviating emotional symptoms leads to improved social functioning, emerging evidence suggests otherwise (Iancu et al., 2020; Ojagbemi, Abiona, Luo, & Gureje, 2018). The dynamic interplay between cognition and depression, and their respective contributions to the restoration of social functioning, remains unclear.

This study has two aims: first, to examine the relationship between cognitive functions (both subjective and objective) and emotional symptoms using a robust sample of patients with MDD; second, to assess the individual impacts of cognitive function and depressive symptoms on social functioning. The first hypothesis of this study is that cognitive function is independent of emotional symptoms. The second hypothesis is that subjective and objective cognitive functions exert different effects on social functioning.

## Methods

### Study design and participants

The Prospective Cohort Study of Depression in China (PROUD) is a nationally representative, multicenter cohort study. Detailed methods of the PROUD study have been introduced in the published protocol (Zhou et al., 2023). This ongoing study was started in January 2022 and will continue till December 2026. Ethical approvals were obtained from Beijing Anding Hospital, Capital Medical University, Beijing, China, and the independent ethics committee overseeing all participating sites. The study has been registered with the Chinese Clinical Trial Registry (https://www.chictr.org.cn/showproj.html?proj=165790, registration number: ChiCTR2200059053). Written informed consents were obtained from all participants. The current study analyzed the data of the PROUD study collected from June 1, 2022, to June 29, 2024, in 18 qualified tertiary hospitals in China located in 14 provinces and cities. A total of 1,376 eligible patients with MDD were included. Inclusion criteria for participants were: (1) Diagnosed with MDD using the Mini-International Neuropsychiatric Interview (MINI); (2) males and females, aged 18–65 years; (3) having a score of 14 or more on the 17-item Hamilton Depression Rating Scale (HAMD-17); (4) having not taken any antidepressant medications for at least 14 days before screening; and (5) will be treated with antidepressant monotherapy. Patients were excluded if they had psychiatric comorbidities or other medical conditions that might interfere with the completion of the study or require the use of medications prohibited in the protocol.

### Clinical and cognitive assessment

Emotional symptoms include both depressive and anxiety symptoms. In this study, depressive symptoms specifically refer to the core features of MDD, characterized by persistent low mood and anhedonia, representing a subset of emotional symptoms (Anderson et al., 2024). A total of seven standardized scales were used in this study to assess depressive and anxiety symptoms, cognitive function, and social functioning.

#### Hamilton depression rating scale (HAMD-17)

The HAMD-17 is a widely used clinical instrument for assessing the severity of depressive symptoms. This 17-item scale evaluates a range of symptoms, including mood, sleep, appetite, guilt, libido, and loss of interest, with each item scored on a 0–4 scale. The total score ranges from 0 to 52, with higher scores indicating more severe depression (Hamilton, 1960). The HAMD-17 showed good reliability (Cronbach’s α = 0.85) and validity in depression populations (Zheng et al., 1988).

#### Quick inventory of depressive symptomatology-self-report (QIDS-SR16)

The QIDS-SR16 is a self-report instrument that evaluates the severity of depressive symptoms. It includes 16 items covering sleep, appetite, interest, energy, physical symptoms, and mood, with items scored according to frequency of occurrence (0–3). The total score varies from 0 to 27, with higher scores reflecting greater severity of symptoms (Hamilton, 1959; Tang & Zhang, 1984). The QIDS-SR16 has shown good reliability (Cronbach’s α = 0.73–0.82) and validity in depression populations.

#### Hamilton anxiety rating scale (HAMA)

The HAMA is a 14-item scale rating the severity of anxiety symptoms via two factors: physical and psychological. Each item is rated 0–4 for severity of symptoms. The score range is 0–56; a higher score indicates greater severity of symptoms (Liu et al., 2013; Rush et al., 2003). The HAMA has good internal consistency (Cronbach’s α = 0.74–0.92) and acceptable test–retest reliability (correlation coefficients 0.74–0.97) (Maier, Buller, Philipp, & Heuser, 1988).

#### Generalized anxiety disorder-7 (GAD-7)

The GAD-7 is a 7-item self-report scale designed to assess the severity of anxiety symptoms. Items are rated according to symptom frequency (0–3), with total scores ranging from 0 to 21. Higher scores reflect more severe anxiety (Hidalgo & Sheehan, 2012). The GAD-7 exhibits excellent reliability (Cronbach’s α = 0.82) (Spitzer, Kroenke, Williams, & Löwe, 2006) and has a strong correlation (r = 0.85) with the HAMA (Ruiz et al., 2011) in patients with depression or anxiety.

#### Chinese brief cognitive test (C-BCT)

Cognitive functioning was assessed using the C-BCT, which is based on the MATRICS Consensus Cognitive Battery (MCCB) (Shi et al., 2015). It has been validated in a large-scale study of schizophrenia patients and has shown good internal consistency (Cronbach’s α = 0.75) and test–retest reliability (ICC = 0.62–0.76) (Ye et al., 2022). Also, additional studies in both the depression (Zhou et al., 2023) and the schizophrenia populations (Du et al., 2024; Zhou et al., 2024; Zhu et al., 2024) provide further evidence of its reliability and validity supporting the robustness of this scale for assessing cognitive function in these clinical groups.

#### Perceived deficits questionnaire-depression-5 item (PDQ-D5)

The PDQ-D5 assesses patients’ self-perceived cognitive deficits. It includes 5 items rated on the severity of cognitive symptoms (0–4), with a total score ranging from 0 to 20. Higher scores indicate greater perceived cognitive dysfunction (Sullivan, Edgley, & Dehoux, 1990). The PDQ-D5 has demonstrated good reliability (Cronbach’s α = 0.795–0.948) and validity in depressed populations (Shi et al., 2017).

#### Sheehan disability scale (SDS)

The SDS evaluates the impact of depression on a patient’s work, social life, and family responsibilities. The total score ranges from 0 to 30, with higher scores indicating greater functional impairment (Sheehan et al., 2011; Sheehan, Harnett-Sheehan, & Raj, 1996 ^)^. The SDS has demonstrated strong reliability (Cronbach’s α = 0.94) and validity in depressed populations (Leu et al., 2015).

### Procedures

Clinic visits for all patients took place at study sites at baseline and Weeks 8 and 52. The interviews were conducted by interviewers with standardized training. The training included practice scoring with feedback from an expert group and one-on-one discussion with the raters who scored very differently from the others. The C-BCT was administered using a tablet at baseline and Weeks 8 and 52. The tasks were administered in the following order: Trail Making Test, Part A (TMT-A), Symbol Coding, Continuous Performance Test (CPT), and Digit Span. The entire assessment would take place in a quiet room free from distractions, with only the researcher and the patient present. All patients were encouraged to make their best effort to complete the tasks and were allowed to take breaks if they felt tired or uncomfortable.

### Statistical analysis

The prospective relationships between depressive symptoms and cognitive function were analyzed using cross-lagged panel modeling (CLPM). This method was chosen to systematically examine the directional effects and to offer insights into the temporal associations between these two variables. The analysis specifically aimed to address two key questions: (1) whether depressive symptoms and cognitive function mutually influence each other over time, and (2) the direction of these influences – whether cognitive function predicts subsequent depressive symptoms or vice versa. No constraints were applied to the CLPM in this study. To handle the missing data, we employed full information maximum likelihood (FIML) estimation, which directly fits the models to the raw data. This approach is recognized for producing less biased and more reliable results compared with traditional methods such as listwise deletion (Orth, Clark, Donnellan, & Robins, 2021). Standardized estimates and 95% confidence intervals were reported for the paths of interest. Model fit was evaluated using the comparative fit index (CFI) and root mean square error of approximation (RMSEA), where a CFI near one and an RMSEA close to zero indicated a good model fit. In all models, benchmark values for interpreting the size of the CLPM cross-lag effect, standardized betas, may be interpreted similarly to correlation coefficients wherein small = 0.1, medium = 0.3, and large = 0.5 (Bredemeier et al., 2023; Cohen, 1992).

To ensure the robustness of our findings, we conducted a series of sensitivity analyses. First, to strengthen the findings, we employed both self-report and clinician-rated tools for evaluations in the current study (HAMD-17 and QIDS-SR16 for depressive symptoms, HAMA and GAD-7 for anxiety symptoms, and C-BCT and PDQ-D5 for cognitive function). Second, we applied a multiple-group CLPM, stratified by episode status (first episode vs. relapse) and treatment type (SSRIs vs. other medications). Third, to explore the contribution of key predictors to changes in symptoms and cognitive function, we included variables such as age, total illness duration, education level, and number of episodes based on a literature review and clinical experience (Hasselbalch, Knorr, & Kessing, 2011; Sachs-Ericsson et al., 2013; Zaninotto et al., 2016), to assess their impact on both domains. Finally, data from three follow-up time points were included to further validate the relationship between symptoms and cognitive function in the model. The 52-week follow-up data included all participants who successfully completed the follow-up, without any specific inclusion or exclusion criteria.

Linear mixed models (LMM) were constructed using all available data without imputation. The models included random intercepts, with visit time modeled as fixed effects and participants as random effects, to estimate linear changes in cognitive function and depressive symptoms over the assessment waves. This approach was chosen due to the limitations of the cross-lagged panel model (CLPM) in explicitly modeling longitudinal trends in outcome measures. Consequently, assessing the extent of longitudinal change is essential for determining the appropriateness of applying these models in this context (Best & Cosco, 2022).

A multiple linear regression analysis was conducted to evaluate the contribution of changes in depressive symptoms and cognitive function to the improvement in social functioning. The model’s covariates included the number of depressive episodes, total illness duration (in months), and age. To assess multicollinearity among the variables, the variance inflation factor (VIF) was calculated, with a threshold of 2 applied to identify potential collinearity issues.

All CLPM were conducted in Mplus version 8.1, and other analyses were performed using SAS for Windows, version 9.4 (SAS Institute, Cary, NC) or R version 4.3.2 (R Foundation for Statistical Computing, Vienna, Austria).

## Results

### Demographic and clinical characteristics

Among the 1,376 patients included in the study, 900 (65.41%) were female and 252 (18.37%) had a family history of mental disorders. A total of 820 patients (59.85%) were experiencing their first episode, and 722 (52.47%) were treated with SSRIs. The median duration of illness was 12.00 months (interquartile range: 2.00–49.00 months), and the median age was 27.86 years (interquartile range: 22.88–35.86 years) (Table 1). Further stratified analyses of basic demographic and clinical characteristics by episode status (first episode vs. relapse) and treatment type (SSRIs vs. other medications) are presented in Supplementary eTable 1 and Supplementary eTable 2, respectively.

**Table 1.** Demographic and clinical characteristics of the participants

Variables	*n* (%) or median (interquartile range)
Sex	
Male	476 (34.59)
Female	900 (65.41)
Family history of mental disorder	252 (18.37)
Body mass index category	
Normal or healthy weight	673 (48.91)
Overweight	361 (26.24)
Underweight	342 (24.85)
Ethnicity	
Han	1279 (93.91)
Hui	26 (1.91)
Manchu	12 (0.88)
Mongolian	9 (0.66)
Other	36 (2.64)
Education level	
Primary school	26 (1.91)
Middle school	121 (8.90)
High school	177 (13.02)
University	866 (63.72)
Postgraduate	169 (12.44)
Geographic location	
Urban	1112 (84.76)
Rural	200 (15.24)
Monthly income range (RMB)	
Below 1,000	35 (2.78)
1,001–5,000	365 (29.04)
5,001–10,000	466 (37.07)
Above 10,000	391 (31.11)
Type of health insurance	
Urban resident basic medical insurance	298 (23.28)
Urban employee basic medical insurance	586 (45.78)
Public healthcare	68 (5.31)
Full self-pay	123 (9.61)
New rural cooperative medical insurance	180 (14.06)
Other	25 (1.95)
Marital status	
Single	828 (61.06)
Married	448 (33.04)
Divorced/widowed	80 (5.90)
Employment status	
Part-time job	44 (3.27)
Full-time job	662 (49.18)
Student	350 (26.00)
Retired/unemployed	290 (21.55)
First episode	820 (59.85)
Type of medication	
SSRIs	722 (52.47)
Other	513 (47.53)
Age (years)	27.86 (22.88–35.86)
Overall duration of illness (months)	12.00 (2.00–49.00)
Duration of the current episode (months)	2.00 (1.00–8.00)
Onset age (year)	27.00 (21.00–35.00)
Number of episodes	1.00 (1.00–2.00)

*Note:* RMB, Renminbi; SSRIs, Selective Serotonin Reuptake Inhibitors. Percentages are calculated from the total sample size (*n* = 1376). Data presented as *n* (%) or median (interquartile range).

### Trends in symptom changes over time

Supplementary eFigure 1 in the supplement summarizes the trend of changes in total scores across different measurements. LMM revealed that the changes from baseline to Week 8 were statistically significant. Specifically, the C-BCT total score increased from 48.82 at baseline to 53.85 at Week 8 (F = 94.68, *P* < 0.001); the HAMD-17 total score decreased from 20.72 at baseline to 10.53 at Week 8 (F = 1625.93, *P* < 0.001); the PDQ-D5 total score decreased from 11.30 at baseline to 6.97 at Week 8 (F = 252.52, *P* < 0.001); the SDS total score decreased from 15.31 at baseline to 8.52 at Week 8 (F = 287.52, *P* < 0.001). See the Supplementary Materials for details.

### Relationships between cognitive function, depressive symptoms, and anxiety symptoms

**Figure 1.**
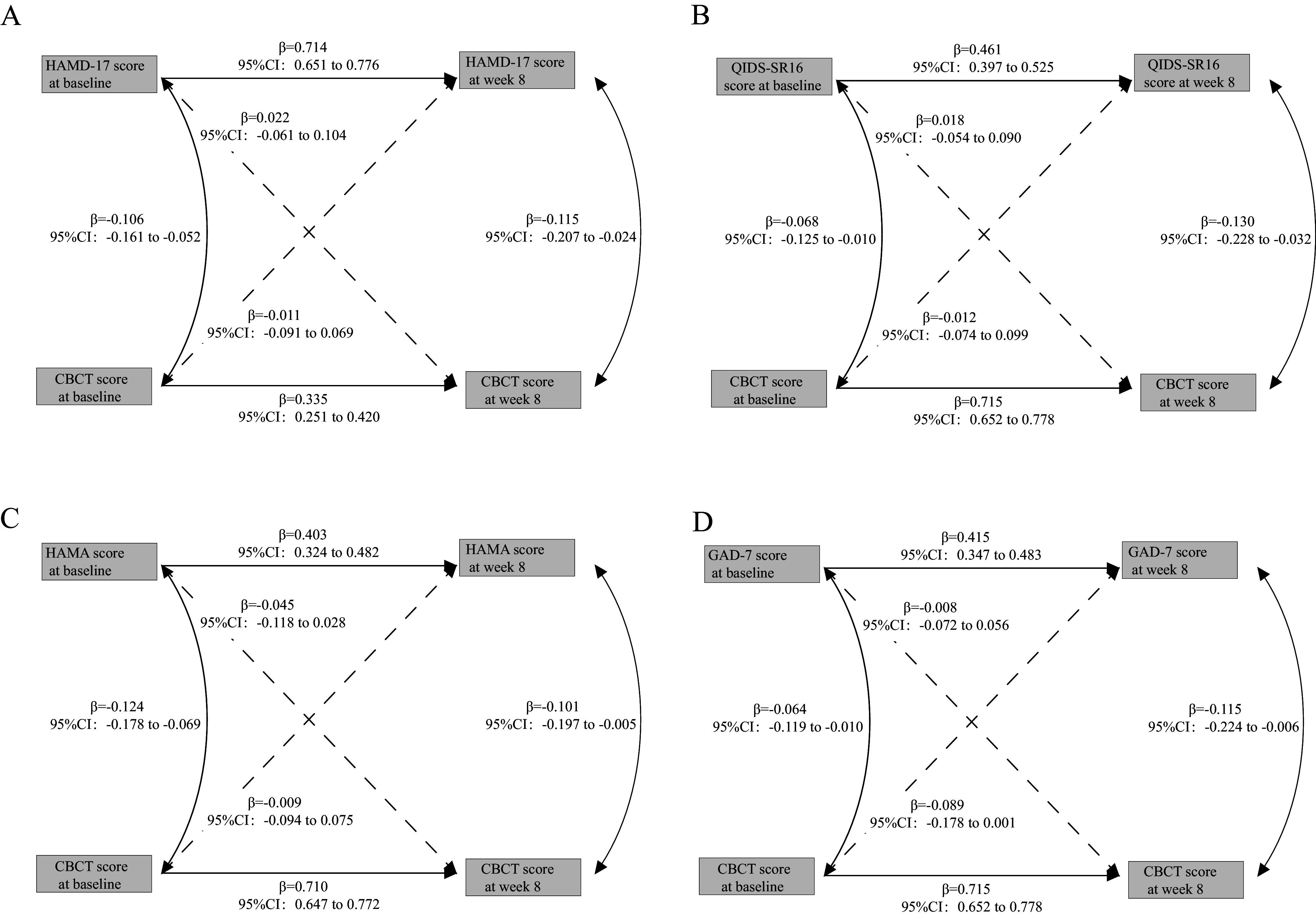
Cross-lagged panel models depicting the associations of objective cognition with depressive symptoms and with anxiety symptoms. *Note:* Standardized estimates with 95% confidence intervals are presented. Solid lines in the Cross-Lagged Panel Models indicate statistically significant standardized estimates, while dashed lines represent estimates that are not statistically significant. A total of 504 patients were followed at Week 8.

Results of the cross-lagged panel models depicting the relationships of objective cognition with depressive symptoms and with anxiety symptoms are displayed in Figure 1. As expected, all cross-sectional relationships between the variables were statistically significant at both time points (*P* < 0.05). In the analysis of prospective associations, statistically significant positive relationships were observed between the variables at baseline and Week 8 (*P* < 0.05). However, no significant association was found between depressive symptoms at baseline (measured by HAMD-17 or QIDS-SR16) and cognitive function at Week 8, as measured by C-BCT (*P* > 0.05). Similarly, anxiety symptoms at baseline were not statistically associated with cognitive function at Week 8 measured by C-BCT (*P* > 0.05). All model fit indices were consistent and satisfactory, with RMSEA = 0, CFI = 1.0.

**Figure 2.**
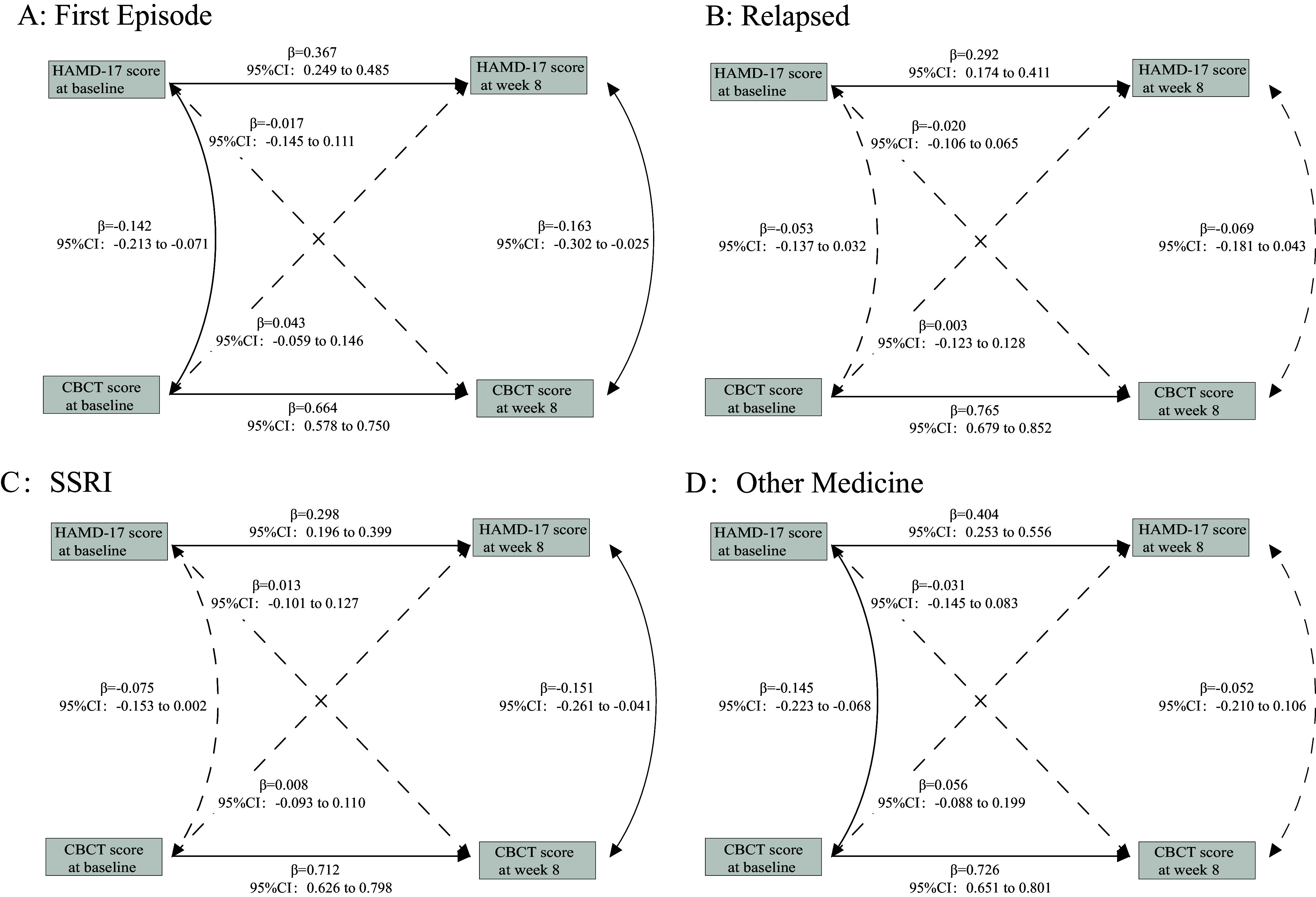
Cross-lagged panel models associations between depressive symptoms and objective cognition stratified by episode groups and treatments. *Note:* Standardized estimates with 95% confidence intervals are presented. Solid lines in the Cross-Lagged Panel Models indicate statistically significant standardized estimates, while dashed lines represent estimates that are not statistically significant. Patients taking medications other than SSRIs included 141 cases on SNRIs (Serotonin-Norepinephrine Reuptake Inhibitors), 23 cases on Mirtazapine, and 318 cases on other antidepressants, such as Bupropion and Trazodone. Additionally, 518 patients were on other psychiatric medications, including 191 on sedative-hypnotics, 348 on benzodiazepines, and 132 on non-benzodiazepines. Some patients were concurrently taking multiple medications.

Further sensitivity analyses exploring the relationship between depressive symptoms and objective cognition were stratified by episode status (first episode versus relapse) and treatment type (SSRIs vs. other medications), as illustrated in Figure 2.

**Figure 3.**
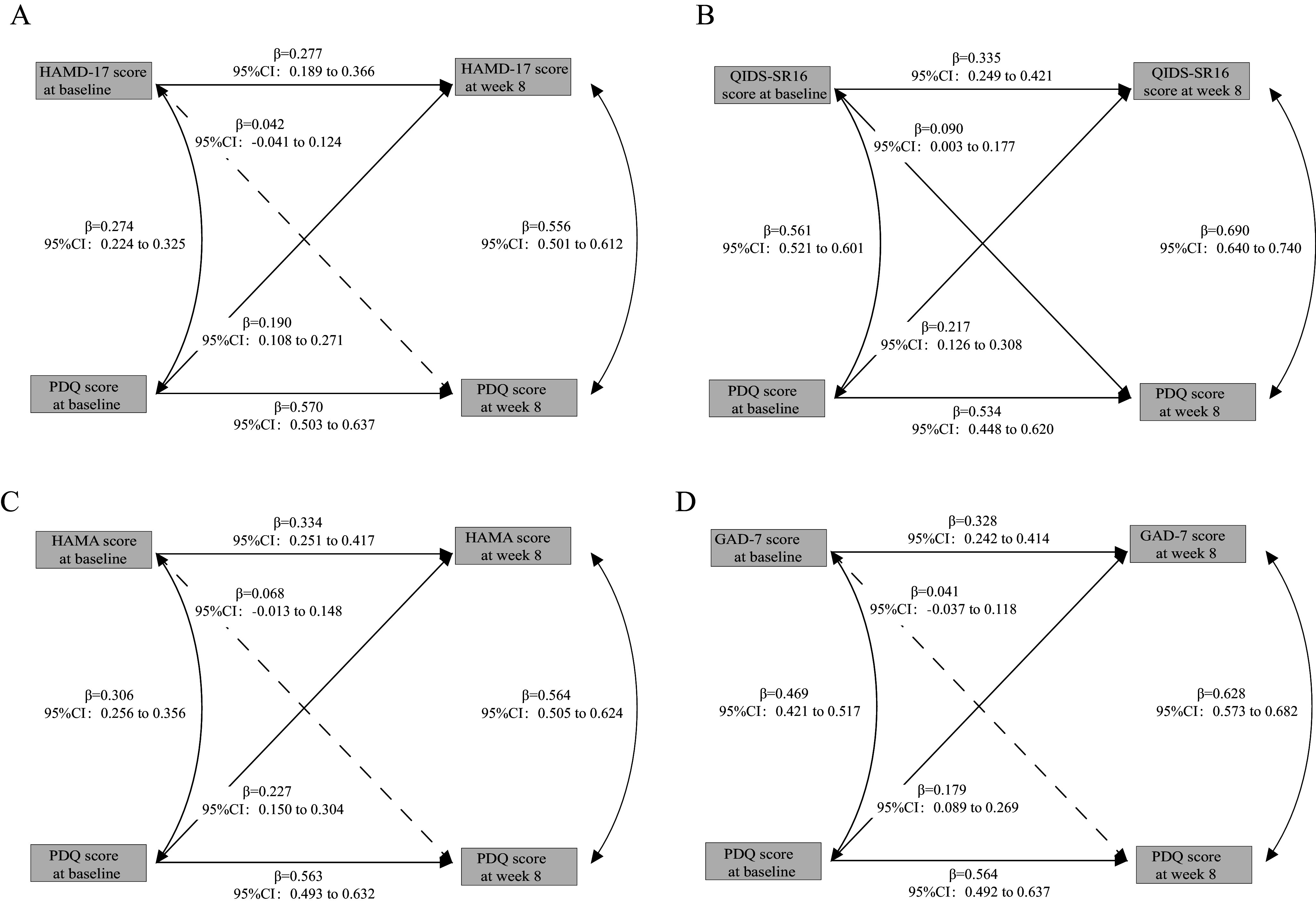
Cross-lagged panel models associations between depressive symptoms and subjective cognition stratified by episode groups and treatment types. *Note:* Standardized estimates with 95% confidence intervals are presented. Solid lines in the Cross-Lagged Panel Models indicate statistically significant standardized estimates, while dashed lines represent estimates that are not statistically significant. Patients taking medications other than SSRIs included 141 cases on SNRIs (Serotonin-Norepinephrine Reuptake Inhibitors), 23 cases on Mirtazapine, and 318 cases on other antidepressants, such as Bupropion and Trazodone. Additionally, 518 patients were on other psychiatric medications, including 191 on sedative-hypnotics, 348 on benzodiazepines, and 132 on non-benzodiazepines. Some patients were concurrently taking multiple medications.

Consistent with prior results, no significant association was observed in any stratification between baseline depressive symptoms (measured by the HAMD-17) and cognitive function at Week 8, as assessed by the C-BCT (*P* > 0.05). Additionally, both the CLPM model with additional variables, including age, total illness duration, education level, and number of episodes (see Supplementary eFigure 2 and Supplementary eFigure3 in the supplement) and the model built using data from three follow-up time points supported these results (see Supplementary eFigure 4 in the supplement). These findings were further validated with analyses of the results of different C-BCT subtests (see Supplementary eFigure 5 in the supplement). In contrast to these findings, the longitudinal analysis of the relationship between depressive symptoms and subjective cognitive function, as presented in Figure 3, revealed that baseline subjective cognitive function significantly predicted depressive symptoms at Week 8. For instance, PDQ-D5 scores at baseline were significantly associated with HAMD-17 scores at Week 8 (β = 0.190, 95% CI: 0.108–0.271). Similarly, PDQ-D5 scores at baseline were associated with QIDS-SR16 scores at Week 8 (β = 0.217, 95% CI: 0.126–0.308), and QIDS-SR16 scores at baseline were also significantly associated with PDQ-D5 scores at Week 8. Additionally, PDQ-D5 scores at baseline were significantly associated with HAMA scores at Week 8 (β = 0.227, 95% CI: 0.150–0.304), as were GAD-7 scores at baseline (β = 0.179, 95% CI: 0.089–0.269). All model fit indices were consistent and satisfactory, with RMSEA = 0, CFI = 1.0.

### Effects of cognitive function and depressive symptoms on social functioning

Separate multiple linear regression models were constructed to assess the improvement in social functioning based on changes in objective and subjective cognitive function. In Model 1, the restoration of social functioning was found to depend solely on changes in depressive symptoms (β = 0.567, *P* < 0.001), with no significant contribution of objective cognitive function. However, in Model 2, the restoration of social functioning was influenced by both changes in depressive symptoms (β = 0.384, *P* < 0.001) and subjective cognitive function (β = 0.551, *P* < 0.001). Additionally, in Model 2, the R^2^ for the decrease in the PDQ-D5 score was 0.196, higher than the R^2^ for the decrease in the HAMD-17 score, which was 0.075 (Table 2).

**Table 2.** Multiple linear regression analysis for improvement of social function

Variables	Estimation	Standard error	t	*P*	VIF
**Model 1**					
Intercept	1.445	1.32637	1.09	0.2765	0
Increase in the C-BCT score	−0.013	0.03875	−0.34	0.7364	1.02
Decrease in the HAMD–17 score*	0.567	0.05361	10.57	<.0001	1.02
Number of episodes	−0.094	0.21748	−0.43	0.6650	1.28
Overall duration of illness (month)	0.006	0.00670	0.82	0.4113	1.36
Age	−0.057	0.04006	−1.42	0.1561	1.08
**Model 2**					
Intercept	0.836	1.15325	0.72	0.4690	0
Decrease in the PDQ-D5 score*	0.551	0.07053	7.81	<.0001	1.21
Decrease in the HAMD–17 score*	0.384	0.05161	7.44	<.0001	1.22
Number of episodes	−0.111	0.20364	−0.54	0.5862	1.33
Overall duration of illness (month)	0.003	0.00582	0.52	0.6039	1.44
Age	−0.031	0.03493	−0.89	0.3765	1.09

*Note:* C-BCT, The Chinese Brief Cognitive Test; PDQ-D5, Perceived Deficits Questionnaire-Depression 5-item; HAMD-17, the 17-item Hamilton Depression Rating Scale; VIF, Variance Inflation Factor.

* Statistically significant variables (*P* < 0.05).

In Model 2, the R^2^ for the decrease in the PDQ-D5 score was 0.196, and the R^2^ for the decrease in the HAMD-17 score was 0.075.

## Discussion

This study performed a comprehensive, large-scale longitudinal analysis using cross-lagged panel models to examine the temporal relationships between cognitive function and emotional symptoms in patients with MDD, clarifying directional relationships among the symptoms and these symptoms’ impacts on the restoration of social functioning. A key finding was that there were significant directional relationships of both depressive and anxiety symptoms with subjective cognitive function but not with objective cognitive function. Furthermore, improvements in subjective cognitive function significantly contributed to the restoration of social functioning, exceeding the effects of improvements in depressive symptoms, whereas objective cognitive function showed no contribution.

The finding that subjective cognitive function can predict depressive and anxiety symptoms during follow-up was consistent with previous findings. For instance, a 2019 study on Intensive Care Unit (ICU) survivors revealed significant associations between subjective cognitive function and psychological symptoms, including anxiety and depression, at various intervals (Brück et al., 2019). Similarly, a 2020 study on adolescents identified a strong relationship between improvements in subjective cognitive function and alleviation in depressive and anxiety symptoms over 3 months of treatment; also, the worsening of subjective cognitive function was associated with symptom exacerbation (Allott et al., 2020). Additionally, an analysis of data from 800 older adults from the 2021 Whitehall II study revealed a significant association between subjective cognitive complaints and depressive symptoms, both cross-sectionally and longitudinally (Topiwala et al., 2021).

These findings may be explained by cognitive models, particularly Beck’s cognitive theory of depression (Beck, 1967). This theory suggests that cognitive biases, including negative self-assessment and attentional bias, contribute to the cognitive distortions prevalent in depression. Impaired cognitive control in the prefrontal cortex diminishes patients’ ability to inhibit negative emotions, resulting in the sustained activation of negative self-referential schemas. This cycle perpetuates depressive symptoms and exacerbates anxiety through rumination (Disner, Beevers, Haigh, & Beck, 2011). Consequently, subjective cognitive distortions – especially negative self-assessments – may reflect these biased cognitive processes and predict the onset and progression of emotional symptoms (Kube et al., 2020). Therefore, the findings highlight the importance of subjective assessments of cognitive function in clinical practice. Moreover, interventions aimed at enhancing subjective cognitive function, such as cognitive–behavioral therapy (CBT) (Ng et al., 2023) and mindfulness training (van der Velden et al., 2023), may effectively address negative cognitive biases and improve emotional regulation. Cognitive control training, which focuses on tasks designed to enhance cognitive flexibility and executive function, may further assist in managing emotional symptoms (Koster et al., 2017).

At the same time, the finding that there was not a significant relationship between objective cognitive function and emotional symptoms was also consistent with previous findings. For instance, a randomized longitudinal study found that despite significant improvements in depressive symptoms following antidepressant treatment, deficits in key cognitive domains such as attention and verbal memory showed minimal improvement (Shilyansky et al., 2016). Additionally, a meta-analysis demonstrated that 73% of objective cognitive variables – particularly those related to processing speed, selective attention, working memory, verbal learning, and executive function – remained impaired in patients with remitted depression (Semkovska et al., 2019).

We found that the improvement of subjective cognitive function contributed more significantly to social functioning recovery than improvements in depressive symptoms. This finding was also consistent with the findings of recent studies. For instance, a 2020 study found that subjective cognitive impairments had a greater incremental effect on depressive symptoms and functional disability than objective cognitive function (Dhillon et al., 2020). Similarly, the 2021 PERFORM-J study found that subjective cognitive impairments were closely related to poorer psychosocial functioning and quality of life among patients with MDD, whereas objective cognitive function showed no significant correlation with psychosocial functioning or quality of life (Sumiyoshi et al., 2021). Additionally, the 2021 CAN-BIND-1 study revealed that the cognitive self-appraisals were strongly associated with the alleviation of depressive symptoms, better recovery of functioning, and enhancements in quality of life (Rnic et al., 2021). From the perspective of self-efficacy theory, enhancements in subjective cognitive function may boost patients’ self-efficacy, leading to increased engagement in work, family, and social activities, thereby facilitating the restoration of social functioning (Ryan & Deci, 2000; Stanley & Maddux, 1986). Additionally, from a psychosocial perspective, improvements in subjective cognitive function may strengthen patients’ resilience, enhancing their ability to regulate responses to stress and environmental challenges (Stover, Shulkin, Lac, & Rapp, 2024). In contrast, improvements in objective cognitive function may exert less influence on social functioning due to a disconnect between objective assessments and real-world demands (Howieson, 2019).

## Strengths and limitations

This study’s strength lies in its large, multicenter, and representative sample. First, as sex differences have been found in many aspects of MDD (Li et al., 2024), the composition of the study population might influence the study results. One strength of the current study was that the male-to-female ratio was ~1:2, consistent with patterns observed in psychiatric epidemiology studies (Bone, Lewis, & Lewis, 2020). Second, the participants exhibited diverse demographic and clinical characteristics, including varying education levels, employment statuses, marital statuses, illness episodes, and durations, which enhanced the generalizability of the findings. Third, cross-lagged panel models were employed to examine the temporal relationships between cognitive function and emotional symptoms in patients with MDD, providing insights into their directional influences and their impact on the restoration of social functioning. Fourth, cognitive evaluations were conducted in controlled laboratory or standardized settings, enhancing the accuracy and reliability of the evaluations. Nevertheless, several limitations must be considered when interpreting these findings. First, although the study included follow-up data, most were collected over an 8-week period, and there was less data from week 52 available. This might have limited the analysis of long-term changes in cognitive, emotional, and functional outcomes. Second, PDQ-5 and C-BCT differ in their cognitive assessment dimensions. PDQ-5 focuses on functional cognitive experiences, including attention/concentration, prospective/retrospective memory, and planning ability (‘Perceived Deficits Questionnaire – Depression, 5-item (PDQ-D-5)’, 2016; Shi et al., 2017). Its items (e.g. ‘difficulty organizing tasks’) directly reflect subjective cognitive impairments in daily life, which are susceptible to core depressive symptoms such as motivation and negative cognitive bias. In contrast, C-BCT assesses fundamental neurocognitive functions, including information processing speed, working memory, and reasoning/problem-solving (Ye et al., 2022), which are primarily regulated by neurocircuitry efficiency and exhibit weaker dynamic associations with emotional states. Third, although CLPM allows for the examination of temporal predictive relationships between variables, its observational nature inherently limits causal inference. Unmeasured confounders, such as genetic predisposition and heterogeneity in environmental exposures, may still influence the observed associations. Therefore, our findings should be interpreted as reflecting the dynamic interplay between symptoms and cognition rather than definitive causal pathways. Future studies should employ prospective randomized controlled trials (RCTs) to further elucidate the causal relationship between depressive symptoms and cognitive function. Finally, the relatively young median age of our cohort (27.86 years) may limit the generalizability of our findings to older patients with MDD. Cognitive decline in older adults is often intertwined with neurodegenerative processes, potentially altering the relationship between subjective cognitive complaints and emotional symptoms compared with younger individuals. Future research will incorporate age-stratified analyses to assess the generalizability of our conclusions.

## Conclusion

This study revealed that cognitive function predicted reductions in depressive symptoms. Also, subjective cognitive function contributed more significantly to the restoration of social functioning than objective cognitive function, even exceeding the influence of depressive symptoms. These findings highlighted the importance of improving subjective cognitive function to enhance the outcomes of patients with MDD. It is important to develop interventions aimed at achieving full recovery, which are based not only on reducing emotional symptoms but also on improving cognition, especially including patients’ appraisal of their own cognitive functioning.

## Supporting information

Zhou et al. supplementary material Zhou et al. supplementary material

## Supplementary material

The supplementary material for this article can be found at http://doi.org/10.1017/S0033291725001011.

## References

[r1] Allott, K. , Gao, C. , Hetrick, S. E. , Filia, K. M. , Menssink, J. M. , Fisher, C. , … Cotton, S. M. (2020). Subjective cognitive functioning in relation to changes in levels of depression and anxiety in youth over 3 months of treatment. BJPsych Open, 6 (5), e84. 10.1192/bjo.2020.68 32753079 PMC7453798

[r2] Anderson, E. , Crawford, C. M. , Fava, M. , Ingelfinger, J. , Nikayin, S. , Sanacora, G. , … Teel, J. (2024). Depression – understanding, identifying, and diagnosing. The New England Journal of Medicine, 390 (17), e41. 10.1056/NEJMp2310179 38692291

[r3] Beck, A. T. (1967). Depression: Clinical, experimental, and theoretical aspects. Hoeber Medical Division, Harper & Row.

[r4] Best, J. R. , & Cosco, T. D. (2022). An analysis of dynamic, bidirectional associations between memory and verbal fluency with depressive symptoms in middle- and older-aged adults: A cohort study. Journal of Affective Disorders, 318, 400–408. 10.1016/j.jad.2022.09.019 36113688

[r5] Bone, J. K. , Lewis, G. , & Lewis, G. (2020). The role of gender inequalities in adolescent depression. Lancet Psychiatry, 7 (6), 471–472. 10.1016/s2215-0366(20)30081-x 32445675

[r6] Bredemeier, K. , Church, L. D. , Bounoua, N. , Feler, B. , & Spielberg, J. M. (2023). Intolerance of uncertainty, anxiety sensitivity, and health anxiety during the COVID-19 pandemic: Exploring temporal relationships using cross-lag analysis. Journal of Anxiety Disorders, 93, 102660. 10.1016/j.janxdis.2022.102660 36527952 PMC9747232

[r7] Brück, E. , Larsson, J. W. , Lasselin, J. , Bottai, M. , Hirvikoski, T. , Sundman, E. , … Olofsson, P. S. (2019). Lack of clinically relevant correlation between subjective and objective cognitive function in ICU survivors: A prospective 12-month follow-up study. Critical Care, 23 (1), 253. 10.1186/s13054-019-2527-1 31300016 PMC6625117

[r8] Cohen, J. (1992). A power primer. Psychological bulletin, 112(1), 155–159. 10.1037//0033-2909.112.1.155 19565683

[r9] Dhillon, S. , Videla-Nash, G. , Foussias, G. , Segal, Z. V. , & Zakzanis, K. K. (2020). On the nature of objective and perceived cognitive impairments in depressive symptoms and real-world functioning in young adults. Psychiatry Research, 287, 112932. 10.1016/j.psychres.2020.112932 32272334

[r10] Disner, S. G. , Beevers, C. G. , Haigh, E. A. , & Beck, A. T. (2011). Neural mechanisms of the cognitive model of depression. Nature Reviews Neuroscience, 12 (8), 467–477. 10.1038/nrn3027 21731066

[r11] Du, N. , Meng, X. , Li, J. , Shi, L. , & Zhang, X. (2024). Decline in working memory in stable schizophrenia may be related to attentional impairment: Mediating effects of negative symptoms, a cross-sectional study. Neuropsychiatric Disease and Treatment, 20, 149–158. 10.2147/ndt.S447965 38288268 PMC10822768

[r12] Evans, V. C. , Iverson, G. L. , Yatham, L. N. , & Lam, R. W. (2014). The relationship between neurocognitive and psychosocial functioning in major depressive disorder: A systematic review. The Journal of Clinical Psychiatry, 75 (12), 1359–1370. 10.4088/JCP.13r08939 25551235

[r62] GBD 2019 Diseases and Injuries Collaborators (2020). Global burden of 369 diseases and injuries in 204 countries and territories, 1990-2019: a systematic analysis for the Global Burden of Disease Study 2019. Lancet (London, England), 396(10258), 1204–1222. 10.1016/S0140-6736(20)30925-9 33069326 PMC7567026

[r13] Hamilton, M. (1959). The assessment of anxiety states by rating. British Journal of Medical Psychology, 32 (1), 50–55. 10.1111/j.2044-8341.1959.tb00467.x 13638508

[r14] Hamilton, M. (1960). A rating scale for depression. Journal of Neurology, Neurosurgery and Psychiatry, 23 (1), 56–62. 10.1136/jnnp.23.1.56 14399272 PMC495331

[r15] Hasselbalch, B. J. , Knorr, U. , & Kessing, L. V. (2011). Cognitive impairment in the remitted state of unipolar depressive disorder: A systematic review. Journal of Affective Disorders, 134 (1–3), 20–31. 10.1016/j.jad.2010.11.011 21163534

[r16] Hidalgo, R. B. , & Sheehan, D. V. (2012). Generalized anxiety disorder. Handbook of Clinical Neurology, 106, 343–362. 10.1016/B978-0-444-52002-9.00019-X 22608630

[r17] Howieson, D. (2019). Current limitations of neuropsychological tests and assessment procedures. Clinical Neuropsychology, 33 (2), 200–208. 10.1080/13854046.2018.1552762 30608020

[r18] Iancu, S. C. , Wong, Y. M. , Rhebergen, D. , van Balkom, A. , & Batelaan, N. M. (2020). Long-term disability in major depressive disorder: A 6-year follow-up study. Psychological Medicine, 50 (10), 1644–1652. 10.1017/S0033291719001612 31284881

[r19] Koster, E. H. W. , Hoorelbeke, K. , Onraedt, T. , Owens, M. , & Derakshan, N. (2017). Cognitive control interventions for depression: A systematic review of findings from training studies. Clinical Psychology Review, 53, 79–92. 10.1016/j.cpr.2017.02.002 28273486

[r20] Kube, T. , Schwarting, R. , Rozenkrantz, L. , Glombiewski, J. A. , & Rief, W. (2020). Distorted cognitive processes in major depression: A predictive processing perspective. Biological Psychiatry, 87 (5), 388–398. 10.1016/j.biopsych.2019.07.017 31515055

[r21] Leu, S. H. , Chou, J. Y. , Lee, P. C. , Cheng, H. C. , Shao, W. C. , Hsien, W. L. , … Chen, V. C. (2015). Validity and reliability of the Chinese version of the Sheehan Disability Scale (SDS-C). Asia-Pacific Psychiatry, 7 (2), 215–222. 10.1111/appy.12182 25847187

[r22] Li, Z. Z. , Chen, Y. P. , Zang, X. C. , Zheng, D. , Lang, X. E. , Zhou, Y. J. , … Zhang, X. Y. (2024). Sexual dimorphism in the relationship between BMI and recent suicidal attempts in first-episode drug-naïve patients with major depressive disorder. Military Medical Research, 11 (1), 66. 10.1186/s40779-024-00572-1 39327593 PMC11425971

[r23] Liu, J. , Xiang, Y. T. , Wang, G. , Zhu, X. Z. , Ungvari, G. S. , Kilbourne, A. M. , … Chiu, H. F. (2013). Psychometric properties of the Chinese versions of the Quick Inventory of Depressive Symptomatology – Clinician Rating (C-QIDS-C) and Self-Report (C-QIDS-SR). Journal of Affective Disorders, 147 (1–3), 421–424. 10.1016/j.jad.2012.08.035 22995944

[r24] Maier, W. , Buller, R. , Philipp, M. , & Heuser, I. (1988). The hamilton anxiety scale: Reliability, validity and sensitivity to change in anxiety and depressive disorders. Journal of Affective Disorders, 14 (1), 61–68. 10.1016/0165-0327(88)90072-9 2963053

[r25] Miller, W. R. (1975). Psychological deficit in depression. Psychological Bulletin, 82 (2), 238–260. 10.1037/h0076367 1096208

[r26] Ng, M. Y. , DiVasto, K. A. , Gonzalez, N. A. , Cootner, S. , Lipsey, M. W. , & Weisz, J. R. (2023). How do cognitive behavioral therapy and interpersonal psychotherapy improve youth depression? Applying meta-analytic structural equation modeling to three decades of randomized trials. Psychological Bulletin, 149 (9–10), 507–548. 10.1037/bul0000395 38713748

[r27] Ojagbemi, A. , Abiona, T. , Luo, Z. , & Gureje, O. (2018). Symptomatic and functional recovery from major depressive disorder in the Ibadan study of ageing. The American Journal of Geriatric Psychiatry, 26 (6), 657–666. 10.1016/j.jagp.2017.12.011 29426606 PMC6008485

[r28] Oluboka, O. J. , Katzman, M. A. , Habert, J. , McIntosh, D. , MacQueen, G. M. , Milev, R. V. , … Blier, P. (2018). Functional recovery in major depressive disorder: Providing early optimal treatment for the individual patient. The International Journal of Neuropsychopharmacology, 21 (2), 128–144. 10.1093/ijnp/pyx081 29024974 PMC5793729

[r30] Orth, U. , Clark, D. A. , Donnellan, M. B. , & Robins, R. W. (2021). Testing prospective effects in longitudinal research: Comparing seven competing cross-lagged models. Journal of Personality and Social Psychology, 120 (4), 1013–1034. 10.1037/pspp0000358 32730068 PMC7854859

[r31] Perceived Deficits Questionnaire – Depression, 5-item (PDQ-D-5). (2016). In R. S. McIntyre (Ed.), Cognitive impairment in major depressive disorder: Clinical relevance, biological substrates, and treatment opportunities (pp. 253–256). Cambridge University Press.

[r32] Preiss, M. , Kucerova, H. , Stepankova, H. , Sos, P. , & Kawaciukova, R. (2007). Cognitive deficits in unipolar depression during remission – Auditory verbal learning test findings. Psychiatrie, 11, 79–83.

[r33] Rnic, K. , Jung, Y. E. , Torres, I. , Chakrabarty, T. , LeMoult, J. , Vaccarino, A. L. , … Lam, R. W. (2021). Association between discrepancy in objective and subjective cognitive abilities and treatment response in patients with major depressive disorder: A CAN-BIND-1 study report. Journal of Affective Disorders, 295, 1095–1101. 10.1016/j.jad.2021.09.002 34706420

[r34] Rock, P. L. , Roiser, J. P. , Riedel, W. J. , & Blackwell, A. D. (2014). Cognitive impairment in depression: A systematic review and meta-analysis. Psychological Medicine, 44 (10), 2029–2040. 10.1017/S0033291713002535 24168753

[r35] Ruiz, M. A. , Zamorano, E. , García-Campayo, J. , Pardo, A. , Freire, O. , & Rejas, J. (2011). Validity of the GAD-7 scale as an outcome measure of disability in patients with generalized anxiety disorders in primary care. Journal of Affective Disorders, 128 (3), 277–286. 10.1016/j.jad.2010.07.010 20692043

[r36] Rush, A. J. , Trivedi, M. H. , Ibrahim, H. M. , Carmody, T. J. , Arnow, B. , Klein, D. N. , … Keller, M. B. (2003). The 16-item quick inventory of depressive symptomatology (QIDS), clinician rating (QIDS-C), and self-report (QIDS-SR): A psychometric evaluation in patients with chronic major depression. Biological Psychiatry, 54 (5), 573–583. 10.1016/s0006-3223(02)01866-8 12946886

[r37] Ryan, R. M. , & Deci, E. L. (2000). Self-determination theory and the facilitation of intrinsic motivation, social development, and well-being. American Psychologist, 55 (1), 68–78. 10.1037//0003-066x.55.1.68 11392867

[r38] Sachs-Ericsson, N. , Corsentino, E. , Moxley, J. , Hames, J. L. , Rushing, N. C. , Sawyer, K. , … Steffens, D. C. (2013). A longitudinal study of differences in late- and early-onset geriatric depression: Depressive symptoms and psychosocial, cognitive, and neurological functioning. Aging & Mental Health, 17 (1), 1–11. 10.1080/13607863.2012.717253 22934752 PMC3535510

[r39] Semkovska, M. , Quinlivan, L. , O’Grady, T. , Johnson, R. , Collins, A. , O’Connor, J. , … Gload, T. (2019). Cognitive function following a major depressive episode: A systematic review and meta-analysis. Lancet Psychiatry, 6 (10), 851–861. 10.1016/s2215-0366(19)30291-3 31422920

[r40] Serra-Blasco, M. , Torres, I. J. , Vicent-Gil, M. , Goldberg, X. , Navarra-Ventura, G. , Aguilar, E. , … Cardoner, N. (2019). Discrepancy between objective and subjective cognition in major depressive disorder. European Neuropsychopharmacology, 29 (1), 46–56. 10.1016/j.euroneuro.2018.11.1104 30503099

[r41] Sheehan, D. V. , Harnett-Sheehan, K. , & Raj, B. A. (1996). The measurement of disability. International Clinical Psychopharmacology, 11 Suppl 3, 89–95. 10.1097/00004850-199606003-00015 8923116

[r42] Sheehan, D. V. , Harnett-Sheehan, K. , Spann, M. E. , Thompson, H. F. , & Prakash, A. (2011). Assessing remission in major depressive disorder and generalized anxiety disorder clinical trials with the discan metric of the Sheehan disability scale. International Clinical Psychopharmacology, 26 (2), 75–83. 10.1097/YIC.0b013e328341bb5f 21102344

[r43] Shi, C. , Kang, L. , Yao, S. , Ma, Y. , Li, T. , Liang, Y. , … Yu, X. (2015). The MATRICS consensus cognitive battery (MCCB): Co-norming and standardization in China. Schizophrenia Research, 169 (1–3), 109–115. 10.1016/j.schres.2015.09.003 26441005 PMC4916953

[r44] Shi, C. , Wang, G. , Tian, F. , Han, X. , Sha, S. , Xing, X. , & Yu, X. (2017). Reliability and validity of Chinese version of perceived deficits questionnaire for depression in patients with MDD. Psychiatry Research, 252, 319–324. 10.1016/j.psychres.2017.03.021 28314227

[r45] Shilyansky, C. , Williams, L. M. , Gyurak, A. , Harris, A. , Usherwood, T. , & Etkin, A. (2016). Effect of antidepressant treatment on cognitive impairments associated with depression: a randomised longitudinal study. Lancet Psychiatry, 3 (5), 425–435. 10.1016/s2215-0366(16)00012-2 26995298 PMC4860142

[r46] Spitzer, R. L. , Kroenke, K. , Williams, J. B. , & Löwe, B. (2006). A brief measure for assessing generalized anxiety disorder: The GAD-7. Archives of Internal Medicine, 166 (10), 1092–1097. 10.1001/archinte.166.10.1092 16717171

[r47] Srisurapanont, M. , Suttajit, S. , Eurviriyanukul, K. , & Varnado, P. (2017). Discrepancy between objective and subjective cognition in adults with major depressive disorder. Scientific Reports, 7 (1), 3901. 10.1038/s41598-017-04353-w 28634383 PMC5478612

[r48] Stanley, M. A. , & Maddux, J. E. (1986). Self-efficacy theory: Potential contributions to understanding cognitions in depression. Journal of Social and Clinical Psychology, 4 (3), 268–278. 10.1521/jscp.1986.4.3.268

[r49] Stover, A. D. , Shulkin, J. , Lac, A. , & Rapp, T. (2024). A meta-analysis of cognitive reappraisal and personal resilience. Clinical Psychology Review, 110, 102428. 10.1016/j.cpr.2024.102428 38657292

[r50] Sullivan, M. J. , Edgley, K. , & Dehoux, E. (1990). A survey of multiple sclerosis: I. Perceived cognitive problems and compensatory strategy use. Chirurgia, 44 (2), 99–105.

[r51] Sumiyoshi, T. , Watanabe, K. , Noto, S. , Sakamoto, S. , Moriguchi, Y. , Hammer-Helmich, L. , & Fernandez, J. (2021). Relationship of subjective cognitive impairment with psychosocial function and relapse of depressive symptoms in patients with major depressive disorder: Analysis of longitudinal data from PERFORM-J. Neuropsychiatric Disease and Treatment, 17, 945–955. 10.2147/ndt.S288108 33814911 PMC8009536

[r52] Tang, Y. , & Zhang, M. (1984). Hamilton anxiety scale (HAMA). Shanghai Archives of Psychiatry, 2, 64–65.

[r53] Topiwala, A. , Suri, S. , Allan, C. , Zsoldos, E. , Filippini, N. , Sexton, C. E. , … Ebmeier, K. P. (2021). Subjective cognitive complaints given in questionnaire: Relationship with brain structure, cognitive performance and self-reported depressive symptoms in a 25-year retrospective cohort study. The American Journal of Geriatric Psychiatry, 29 (3), 217–226. 10.1016/j.jagp.2020.07.002 32736919 PMC8097240

[r54] van der Velden, A. M. , Scholl, J. , Elmholdt, E. M. , Fjorback, L. O. , Harmer, C. J. , Lazar, S. W. , … Kuyken, W. (2023). Mindfulness training changes brain dynamics during depressive rumination: A randomized controlled trial. Biological Psychiatry, 93 (3), 233–242. 10.1016/j.biopsych.2022.06.038 36328822

[r29] World Health Organization. (2023). Depressive disorder (depression). https://www.who.int/news-room/fact-sheets/detail/depression

[r55] Ye, S. , Xie, M. , Yu, X. , Wu, R. , Liu, D. , Hu, S. , … Shi, C. (2022). The Chinese brief cognitive test: Normative data stratified by gender, age and education, Frontiers in Psychiatry, 13, 933642. 10.3389/fpsyt.2022.933642 35859598 PMC9289100

[r56] Zaninotto, L. , Solmi, M. , Veronese, N. , Guglielmo, R. , Ioime, L. , Camardese, G. , & Serretti, A. (2016). A meta-analysis of cognitive performance in melancholic versus non-melancholic unipolar depression. Journal of Affective Disorders, 201, 15–24. 10.1016/j.jad.2016.04.039 27156095

[r57] Zheng, Y. P. , Zhao, J. P. , Phillips, M. , Liu, J. B. , Cai, M. F. , Sun, S. Q. , & Huang, M. F. (1988). Validity and reliability of the Chinese Hamilton depression rating scale. British Journal of Psychiatry, 152, 660–664. 10.1192/bjp.152.5.660 3167442

[r58] Zhou, J. , Xu, J. , Liu, R. , Qi, H. , Yang, J. , Guo, T. , … Wang, G. (2023). A prospective cohort study of depression (PROUD) in China: Rationale and design. Current Medicine (Cham), 2 (1), 1. 10.1007/s44194-022-00018-7 PMC9826756 36643216

[r59] Zhou, J. , Zhou, J. , Feng, Z. , Feng, L. , Xiao, L. , Chen, X. , … Wang, G. (2024). Identifying the core residual symptom in patients with major depressive disorder using network analysis and illustrating its association with prognosis: A study based on the national cohorts in China. General Hospital Psychiatry, 87, 68–76. 10.1016/j.genhosppsych.2024.01.012 38325144

[r60] Zhou, Q. , Zheng, Y. , Guo, X. , Wang, Y. , Pu, C. , Shi, C. , & Yu, X. (2024). Abnormal hedonic process in patients with stable schizophrenia: Relationships to negative symptoms and social functioning. Schizophrenia Research: Cognition, 38, 100325. 10.1016/j.scog.2024.100325 39263562 PMC11388758

[r61] Zhu, J. , Li, J. , Zhou, L. , Xu, L. , Pu, C. , Huang, B. , … Shi, C. (2024). Eye movements as predictor of cognitive improvement after cognitive remediation therapy in patients with schizophrenia. Frontiers in Psychiatry, 15, 1395198. 10.3389/fpsyt.2024.1395198 38690204 PMC11059054

