# Supplementary material for: A longitudinal analysis of the relationship between emotional symptoms and cognitive function in patients with major depressive disorder
Source: Psychol Med. 2025 May 2;55:e131. doi: 10.1017/S0033291725001011 (PMC12094654; doi:10.1017/S0033291725001011)
Supplement: Zhou et al. supplementary material [file S0033291725001011sup001.docx]

Supplementary materials for manuscript

A Longitudinal Analysis of the Relationship Between Depressive Symptoms and Cognitive Function in Patients with Major Depressive Disorder

Contents:

1. Figure S1 Trend of Total Score Changes Across Different Measures
2. Table S1 Basic information stratified by episode
3. Table S2 Basic information stratified by treatment
4. Figure S2 Cross-Lagged Panel Models Illustrating the Associations Between Depressive Symptoms and Objective Cognition, Adjusted for Covariates
5. Figure S3: Cross-Lagged Panel Models Illustrating the Associations Between Depressive Symptoms and Subjective Cognition, Adjusted for Covariates
6. Figure S4 Cross-Lagged Panel Models Illustrating the Associations Between Depressive Symptoms and Objective Cognition Across Three Follow-Up Time Points
7. Figure S5 Cross-Lagged Panel Models Illustrating the Associations Between Depressive Symptoms and different CBCT cognitive tests


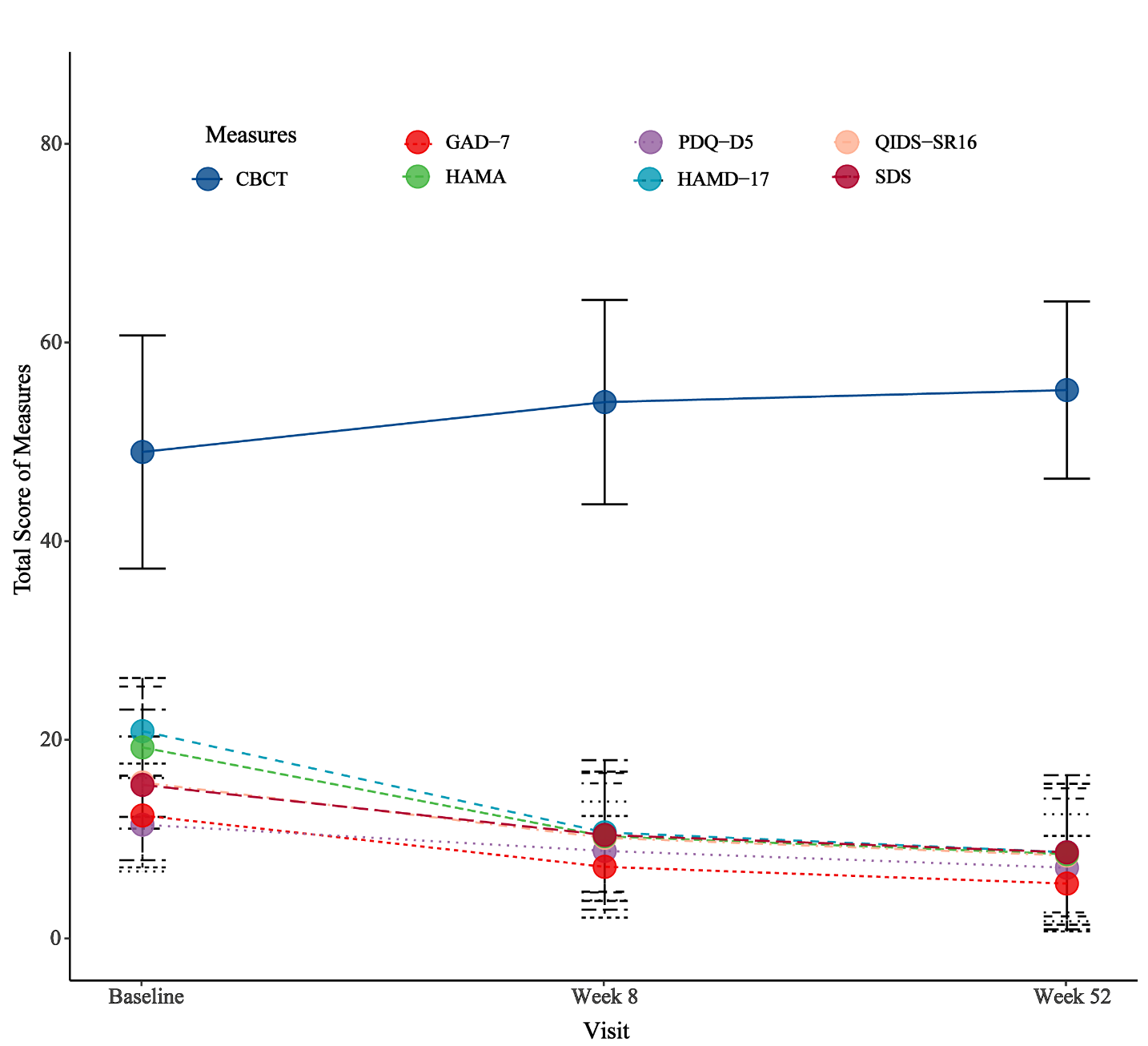


Figure S1. Trend of Total Score Changes Across Different Measures

*Note: A total of 504 patients were followed up at week 8, and 48 patients at week 52.*

Table S1 Basic information stratified by episode

| Variables | Relapsed | First-episode | χ^2^/Z | *P* |
| --- | --- | --- | --- | --- |
| Sex |  |  | 12.954 | 0.0003 |
| Male | 160(29.09) | 316(38.54) |  |  |
| Female | 390(70.91) | 504(61.46) |  |  |
| Family history of mental disorder | 115(20.91) | 137(16.71) | 3.872 | 0.0491 |
| Body Mass Index Category |  |  | 2.763 | 0.2512 |
| Normal or Healthy Weight | 280(50.91) | 393(47.93) |  |  |
| Overweight | 148(26.91) | 213(25.98) |  |  |
| Underweight | 122(22.18) | 214(26.10) |  |  |
| Ethnicity |  |  | 0.974 | 0.9137 |
| Han | 512(93.43) | 765(94.21) |  |  |
| Hui | 11(2.01) | 15(1.85) |  |  |
| Manchu | 5(0.91) | 7(0.86) |  |  |
| Mongolian | 5(0.91) | 4(0.49) |  |  |
| Other | 15(2.74) | 21(2.59) |  |  |
| Education Level |  |  | 4.215 | 0.3777 |
| Middle School | 47(8.61) | 74(9.12) |  |  |
| University | 347(63.55) | 518(63.87) |  |  |
| High School | 77(14.10) | 99(12.21) |  |  |
| Primary School | 14(2.56) | 12(1.48) |  |  |
| Postgraduate | 61(11.17) | 108(13.32) |  |  |
| Geographic Location |  |  | 0.282 | 0.5953 |
| Urban | 455(84.10) | 655(85.18) |  |  |
| Rural | 86(15.90) | 114(14.82) |  |  |
| Monthly Income Range |  |  | 1.399 | 0.7058 |
| Above 10,000 RMB | 170(32.76) | 221(29.99) |  |  |
| Below 1,000 RMB | 14(2.70) | 20(2.71) |  |  |
| 1,001-5,000 RMB | 151(29.09) | 214(29.04) |  |  |
| 5,001-10,000 RMB | 184(35.45) | 282(38.26) |  |  |
| Type of Health Insurance |  |  | 1.808 | 0.8750 |
| Urban Resident Basic Medical Insurance | 121(22.79) | 176(23.56) |  |  |
| Urban Employee Basic Medical Insurance | 242(45.57) | 343(45.92) |  |  |
| Public Healthcare | 27(5.08) | 41(5.49) |  |  |
| Other | 8(1.51) | 17(2.28) |  |  |
| Full Self-Pay | 54(10.17) | 69(9.24) |  |  |
| New Rural Cooperative Medical Insurance | 79(14.88) | 101(13.52) |  |  |
| Marital Status |  |  | 5.309 | 0.0703 |
| Divorced/Widowed | 42(7.69) | 38(4.70) |  |  |
| Single | 329(60.26) | 497(61.51) |  |  |
| Married | 175(32.05) | 273(33.79) |  |  |
| Employment Status |  |  | 4.452 | 0.2166 |
| Housewife/Retired/Unemployed | 132(24.35) | 157(19.58) |  |  |
| Part-Time job | 17(3.14) | 27(3.37) |  |  |
| Full-Time job | 259(47.79) | 402(50.12) |  |  |
| Student | 134(24.72) | 216(26.93) |  |  |
| Type of Medication |  |  | 4.714 | 0.0947 |
| Other | 186(33.82) | 321(39.15) |  |  |
| SNRI (Serotonin-Norepinephrine Reuptake Inhibitors) | 55(10.00) | 86(10.49) |  |  |
| SSRI (Selective Serotonin Reuptake Inhibitors) | 309(56.18) | 413(50.37) |  |  |
| Duration of the current episode(month) | 1.50(0.00-4.00) | 4.00(1.00-12.00) | -9.584 | <.0001 |
| Overall duration of illness(month) | 51.50(27.00-91.00) | 4.00(1.00-12.00) | 25.058 | <.0001 |
| Onset age(year) | 27.00(22.00-35.00) | 27.00(21.00-34.00) | 2.196 | 0.0283 |
| Number of episodes | 2.00(2.00-4.00) | 1.00(1.00-1.00) | 35.581 | <.0001 |

*Note: n (%) or median (interquartile range).*

Table S2 Basic information stratified by treatment

| Variables | SSRIs | Other Medicine | χ^2^/Z | *P* |
| --- | --- | --- | --- | --- |
| Sex |  |  | 0.7759 | 0.3784 |
| Male | 234(35.78%) | 242(33.52%) |  |  |
| Female | 420(64.22%) | 480(66.48%) |  |  |
| Family history of mental disorder | 109(16.77%) | 143(19.81%) | 2.1040 | 0.1469 |
| Body Mass Index Category |  |  | 0.9347 | 0.6267 |
| Normal or Healthy Weight | 311(47.55%) | 362(50.14%) |  |  |
| Overweight | 177(27.06%) | 184(25.48%) |  |  |
| Underweight | 166(25.38%) | 176(24.38%) |  |  |
| Ethnicity |  |  | 2.3361 | 0.6742 |
| Han | 606(94.25%) | 673(93.60%) |  |  |
| Hui | 11(1.71%) | 15(2.09%) |  |  |
| Manchu | 4(0.62%) | 8(1.11%) |  |  |
| Mongolian | 3(0.47%) | 6(0.83%) |  |  |
| Other | 19(2.95%) | 17(2.36%) |  |  |
| Education Level |  |  | 1.6808 | 0.7942 |
| Middle School | 53(8.27%) | 68(9.47%) |  |  |
| University | 413(64.43%) | 453(63.09%) |  |  |
| High School | 88(13.73%) | 89(12.40%) |  |  |
| Primary School | 11(1.72%) | 15(2.09%) |  |  |
| Postgraduate | 76(11.86%) | 93(12.95%) |  |  |
| Geographic Location |  |  | 2.8788 | 0.0898 |
| Urban | 545(86.51%) | 567(83.14%) |  |  |
| Rural | 85(13.49%) | 115(16.86%) |  |  |
| Monthly Income Range |  |  | 2.9101 | 0.4057 |
| Above 10,000 RMB | 179(29.39%) | 212(32.72%) |  |  |
| Below 1,000 RMB | 20(3.28%) | 15(2.31%) |  |  |
| 1,001-5,000 RMB | 185(30.38%) | 180(27.78%) |  |  |
| 5,001-10,000 RMB | 225(36.95%) | 241(37.19%) |  |  |
| Type of Health Insurance |  |  | 10.094 | 0.0726 |
| Urban Resident Basic Medical Insurance | 160(25.76%) | 138(20.94%) |  |  |
| Urban Employee Basic Medical Insurance | 282(45.41%) | 304(46.13%) |  |  |
| Public Healthcare | 25(4.03%) | 43(6.53%) |  |  |
| Other | 13(2.09%) | 12(1.82%) |  |  |
| Full Self-Pay | 64(10.31%) | 59(8.95%) |  |  |
| New Rural Cooperative Medical Insurance | 77(12.40%) | 103(15.63%) |  |  |
| Marital Status |  |  | 0.9391 | 0.6253 |
| Divorced/Widowed | 42(6.55%) | 38(5.31%) |  |  |
| Single | 388(60.53%) | 440(61.54%) |  |  |
| Married | 211(32.92%) | 237(33.15%) |  |  |
| Employment Status |  |  | 0.3775 | 0.9449 |
| Housewife/Retired/Unemployed | 142(22.22%) | 148(20.93%) |  |  |
| Part-Time job | 20(3.13%) | 24(3.39%) |  |  |
| Full-Time job | 312(48.83%) | 350(49.50%) |  |  |
| Student | 165(25.82%) | 185(26.17%) |  |  |
| First Episode | 407(62.81%) | 413(57.20%) | 4.4671 | 0.0346 |
| Duration of the current episode(month) | 2.00(1.00-8.00) | 2.00(1.00-9.00) | 0.6048 | 0.5454 |
| Overall duration of illness(month) | 11.00(2.00-48.00) | 14.00(2.00-53.00) | -1.8117 | 0.0702 |
| Onset age(year) | 27.00(22.00-35.00) | 26.00(21.00-34.00) | 1.2749 | 0.2025 |
| Number of episodes | 1.00(1.00-2.00) | 1.00(1.00-2.00) | -2.0832 | 0.0374 |

*Note: n (%) or median (interquartile range).*


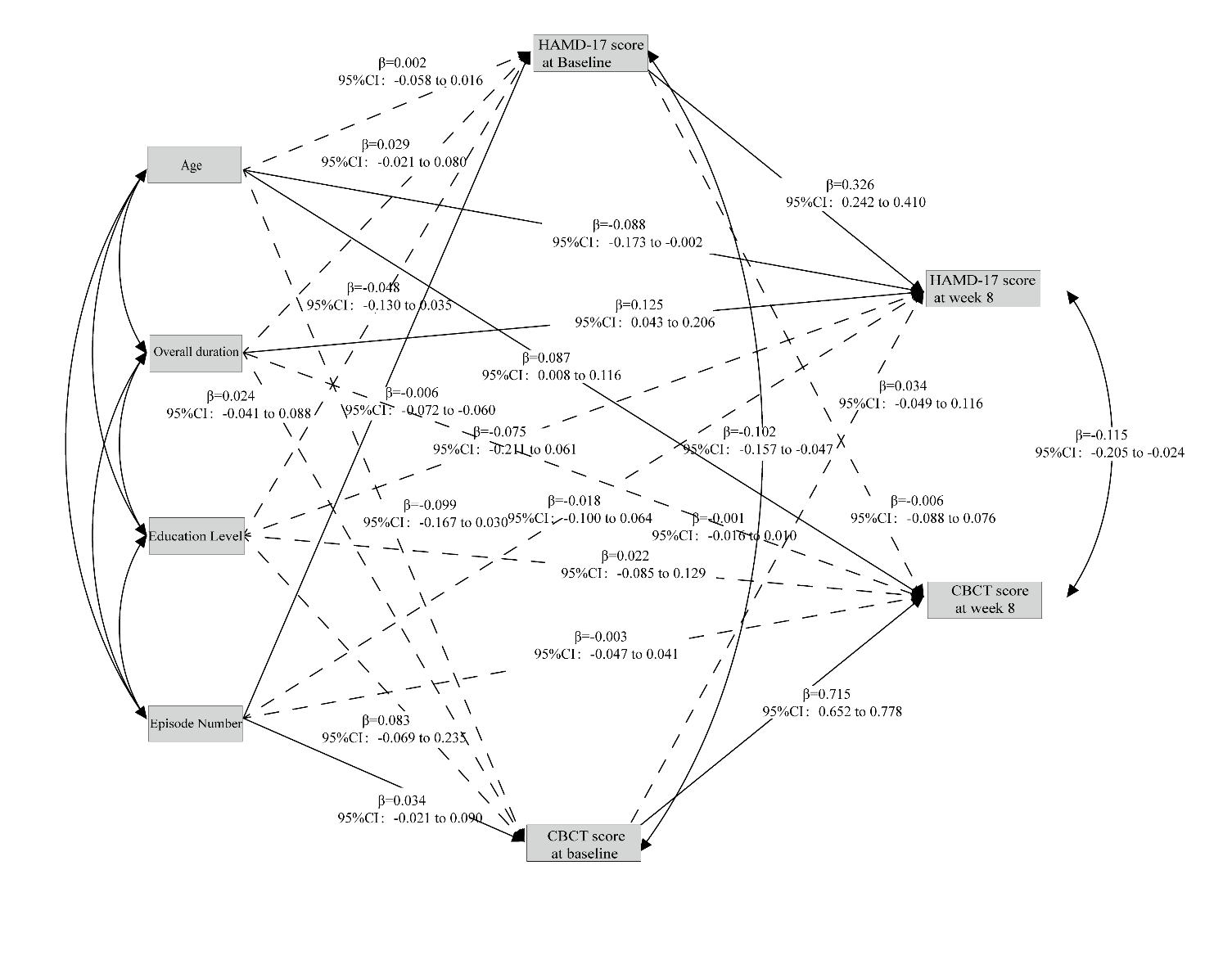


Figure S2: Cross-Lagged Panel Models Illustrating the Associations Between Depressive Symptoms and Objective Cognition, Adjusted for Covariates

*Note: Standardized estimates with 95% confidence intervals are shown. Solid lines in the Cross-Lagged Panel Models indicate statistically significant standardized estimates, while dashed lines represent estimates that are not statistically significant. A total of 504 patients were followed at week 8.*


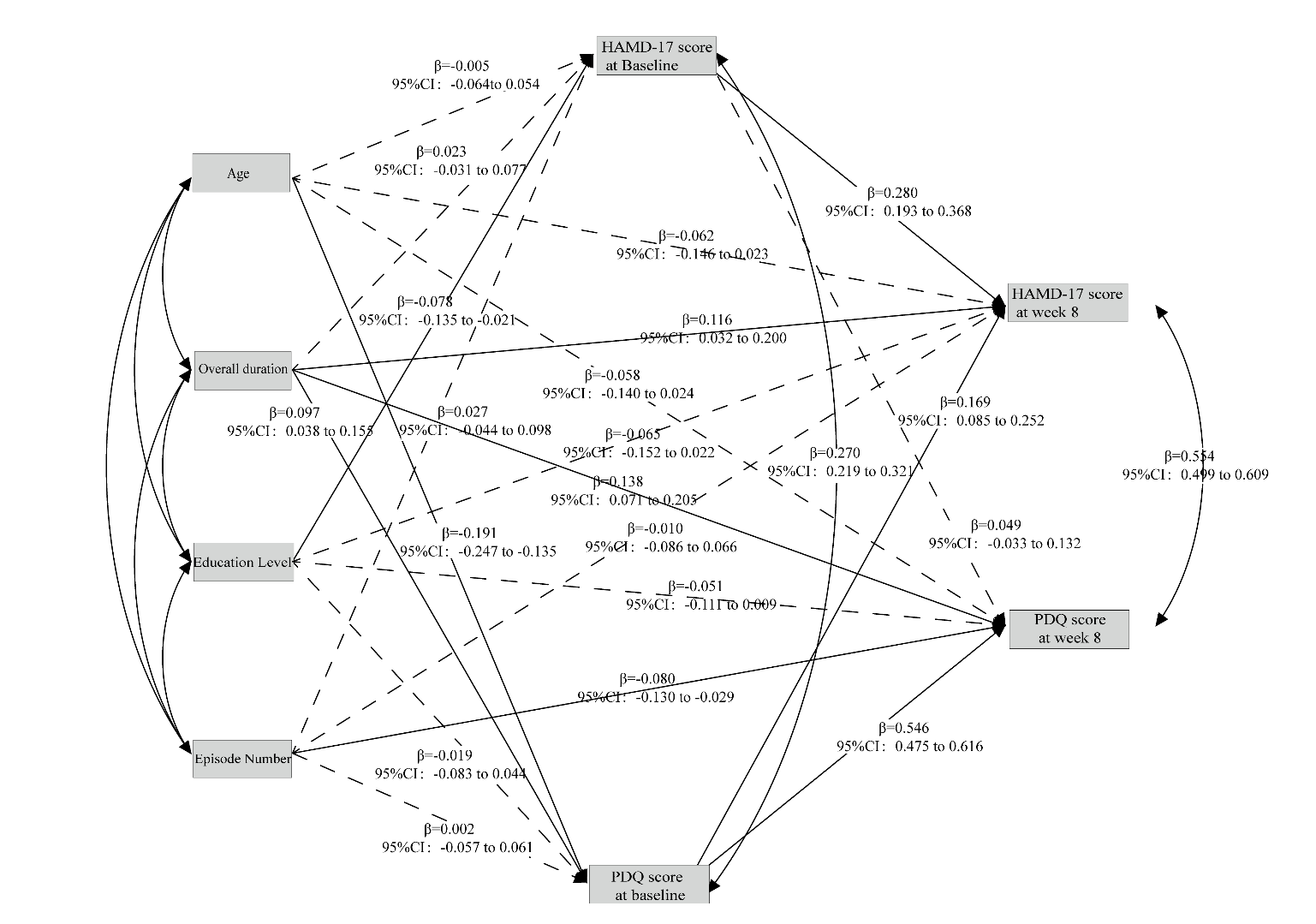


Figure S3: Cross-Lagged Panel Models Illustrating the Associations Between Depressive Symptoms and Subjective Cognition, Adjusted for Covariates

*Note: Standardized estimates with 95% confidence intervals are shown. Solid lines in the Cross-Lagged Panel Models indicate statistically significant standardized estimates, while dashed lines represent estimates that are not statistically significant. A total of 504 patients were followed at week 8.*


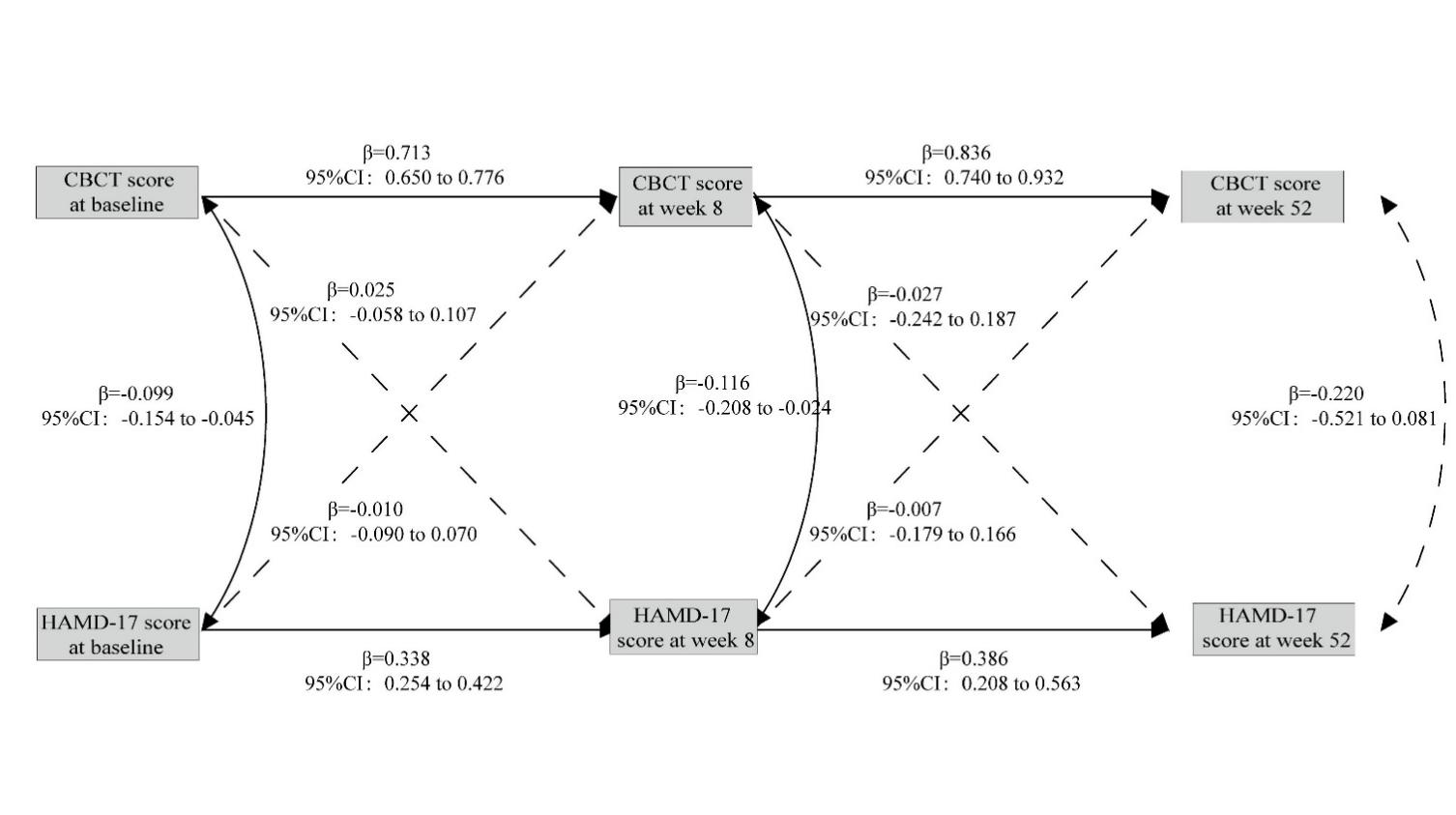


Figure S4: Cross-Lagged Panel Models Illustrating the Associations Between Depressive Symptoms and Objective Cognition Across Three Follow-Up Time Points

*Note: Standardized estimates with 95% confidence intervals are shown. Solid lines in the Cross-Lagged Panel Models indicate statistically significant standardized estimates, while dashed lines represent estimates that are not statistically significant. A total of 504 patients were followed up at week 8, and 48 patients at week 52.*


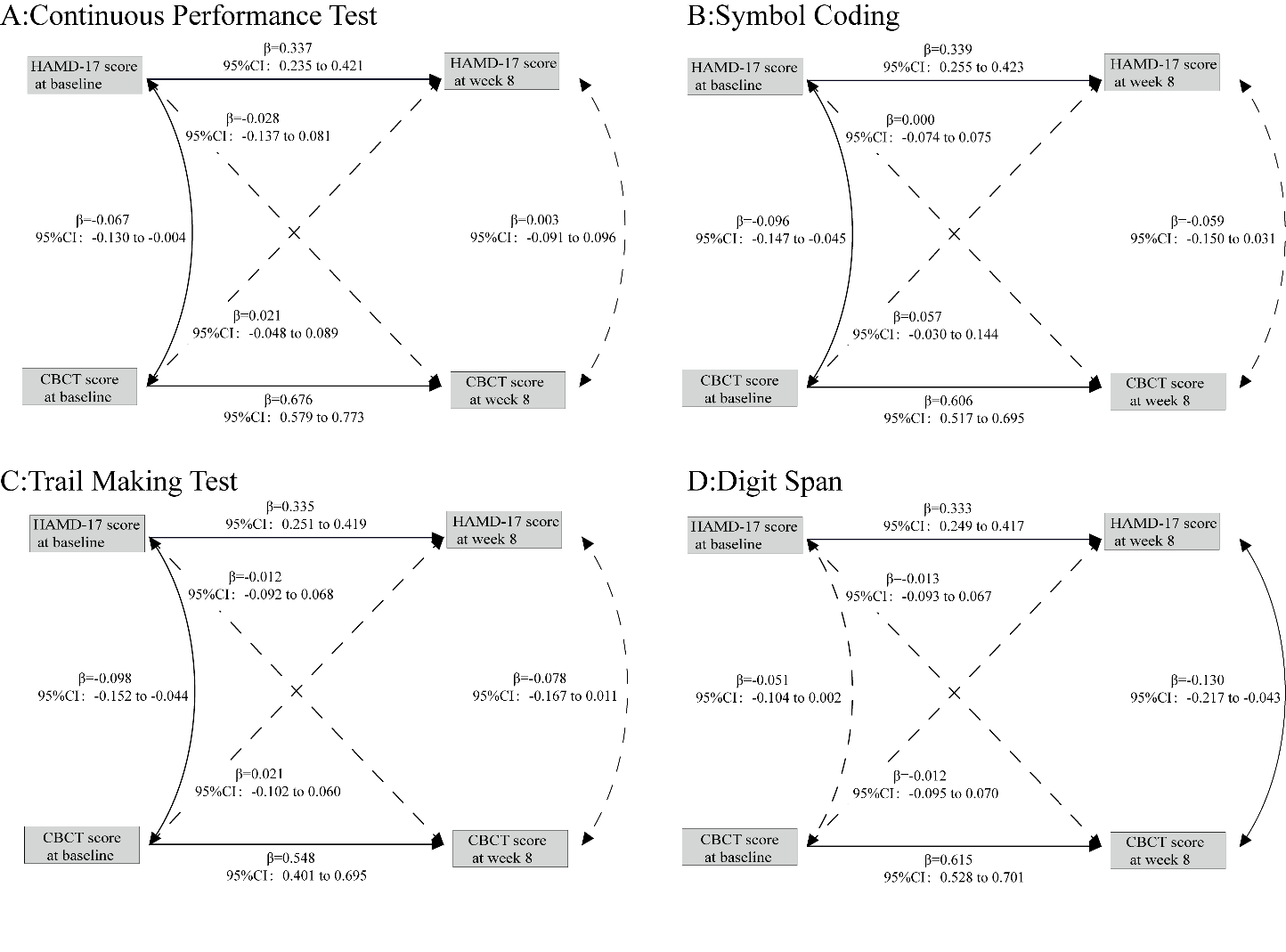
Figure S5 Cross-Lagged Panel Models Illustrating the Associations Between Depressive Symptoms and different CBCT cognitive tests.

*Note: Standardized estimates with 95% confidence intervals are shown. Solid lines in the Cross-Lagged Panel Models indicate statistically significant standardized estimates, while dashed lines represent estimates that are not statistically significant. A total of 504 patients were followed at week 8.*
